# Bioinspired Photoreceptors with Neural Network for Recognition and Classification of Sign Language Gesture

**DOI:** 10.3390/s23249646

**Published:** 2023-12-06

**Authors:** Claudio Urrea, John Kern, Ricardo Navarrete

**Affiliations:** Electrical Engineering Department, Faculty of Engineering, University of Santiago of Chile, Las Sophoras 165, Estación Central, Santiago 9170020, Chile; john.kern@usach.cl (J.K.); ricardo.navarretec@usach.cl (R.N.)

**Keywords:** photoreceptor cells, retina bioinspired, sign recognition, deep learning, MediaPipe library

## Abstract

This work addresses the design and implementation of a novel PhotoBiological Filter Classifier (PhBFC) to improve the accuracy of a static sign language translation system. The captured images are preprocessed by a contrast enhancement algorithm inspired by the capacity of retinal photoreceptor cells from mammals, which are responsible for capturing light and transforming it into electric signals that the brain can interpret as images. This sign translation system not only supports the effective communication between an agent and an operator but also between a community with hearing disabilities and other people. Additionally, this technology could be integrated into diverse devices and applications, further broadening its scope, and extending its benefits for the community in general. The bioinspired photoreceptor model is evaluated under different conditions. To validate the advantages of applying photoreceptors cells, 100 tests were conducted per letter to be recognized, on three different models (V1, V2, and V3), obtaining an average of 91.1% of accuracy on V3, compared to 63.4% obtained on V1, and an average of 55.5 Frames Per Second (FPS) in each letter classification iteration for V1, V2, and V3, demonstrating that the use of photoreceptor cells does not affect the processing time while also improving the accuracy. The great application potential of this system is underscored, as it can be employed, for example, in Deep Learning (DL) for pattern recognition or agent decision-making trained by reinforcement learning, etc.

## 1. Introduction

Communication is fundamental in any group activity, as it is the means for giving instructions or sharing information (messages) from a subject (sender) to another (receiver) through a channel. Humans use language as an instrument to materialize communication, which can be spoken or written. Spoken language allows us to communicate immediately, while communication through written language will depend on the time it takes readers to receive and understand information. However, problems arise when communication is not effective, i.e., when the sender, the receiver, the channel, or even the message suffer alterations while traveling through the channel. These situations occur when people suffer an alteration or have reduced capacity to send a message—speech problems—or receive a message—receiving problems, in which the ear is commonly involved. According to the United Nations, it is estimated that 70 million deaf people in the world use, as a group, 300 different sign languages to achieve successful communication [1]. Despite the large number of people with hearing impairments, there is no cross-cultural learning culture regarding sign language, but several investigations have presented technological alternatives to facilitate real-time gesture interpretations and translations [2,3,4,5].

Thanks to the evolution of computers, deep learning is the preferred method for researchers [6,7,8,9,10,11,12,13]. This is due to the good results as a classifier or pattern identifier, but accuracy and response times continue to be variables to optimize. In addition, when implementing, the environment’s lighting must be considered to achieve the best possible image with details to process. Different investigations indicate that attacking a problem from a bioinspired point of view allows improving overall results, as in the case of [14,15] which apply a bioinspired model for processing environmental images so that an unmanned vehicle trained with reinforcement learning can move and obtain better overall performance compared to traditional training.

Common image processing usually uses standard filters or kernels that allow highlighting areas, binarizing, filtering contours, smoothing images, etc. These kernels are convolved with the input image to obtain a filtered output. On the other hand, bioinspired models generate image processing focused on how a biological cell would accomplish it. That is, they have a response based on neuronal excitation and, analogously to the already known kernels, they are able to regulate lighting, extract independent colors, detect movement, highlight contours, etc.

Retinal bioinspired models work in the frequency domain and can be generalized as a set of high, low, or band pass filters, depending on the biological cell used. In addition, addressing image processing from a bioinspired perspective allows highlighting elements of a scene that are often suppressed when convolving the scene with a common kernel. Thus, incorporating biological cells into neural networks manages to improve the accuracy percentage, compared to the model without biological cells.

Therefore, this work proposes the application of bioinspired photoreceptor cells as a complementary alternative to deep learning models. It should be noted that deep learning models are usually trained with a set of images obtained directly from a camera, but these can be improved or preprocessed in order to present as much detail as possible and consequently improve the error rate of the neural system, as proposed in this work. In this case, the application of bioinspired cells is limited to photoreceptor type cells, since they are responsible for regulating the illumination of the scene.

Specifically, the global architecture consists of four fundamental stages: the first is the self-regulation of the scene lighting by means of a bioinspired photoreceptor cell model, the second corresponds to hand detection anywhere in the scene, the third corresponds to the segmentation of the Region Of Interest (ROI), and the fourth corresponds to the classification of the gesture detected in the ROI.

The tests are carried out on a neural network capable of classifying the alphabet of the American alphabet that has been trained with three different datasets, which come from the same dataset containing 87,000 images [16]. That is, two additional datasets are generated, which will allow training three different models, the first being trained with the dataset without prior processing, the second model is trained with images processed by bioinspired photoreceptor cells, and the third model is trained with a dataset obtained from the images processed by photoreceptor cells and subjected to the MediaPipe library.

The contribution of this research can be summarized in the following points:

(1) Addressing a classification problem from a biological retinal perspective;
(2) Development of a bioinspired architecture that self-regulates the dynamic contrast of a scene and improves system performance.

This work is structured as follows: Section 1 discusses the presentation of our novel photobiological filter classifier for hand gesture classification; Section 2 addresses a review of the state of the art associated with works related to our research proposal and how bioinspired models and Deep Learning allow us to advance towards a new frontier in gesture recognition; Section 3 details the structure of the photobiological classifier filter, highlighting its innovative approach to recognition with MediaPipe and a bioinspired model and presents the performance of three models (V1, V2, and V3); Section 4 provides a quantitative analysis of the model, the implementation of the bioinspired photoreceptor, the results obtained in the stage of ASL-alphabet letter recognition, some comparative analysis, and performance tests under different training networks; Section 5 presents the limitations of the system and its adaptation to lighting conditions; Section 6 deals with the conclusions and the great application potential of this research; and, lastly, Section 7 presents future work projections.

## 2. Related Work

Sign language recognition is a crucial tool for improving the quality of life of people with hearing disabilities. There are over 200 different sign languages currently in use, with languages like American Sign Language (ASL), British Sign Language (BSL), and French Sign Language (LSF) being among the most widely used [10].

In recent years, there has been great interest in the use of artificial intelligence techniques, specifically of CNNs for sign language recognition [17,18,19]. Below, works related to the study of sign language assisted by artificial intelligence are presented, as well as studies that implement bioinspired retina models.

The efforts for finding a way to translate sign language are not recent; in fact, already in 2012, news was presented in media outlets like BBC, with the first applications able to translate sign language on smartphones with a camera [20]. Since then, thanks to the accelerated evolution of processors, multiple endeavors with varied success rates have been introduced. In addition, after the incorporation of Deep Learning (DL), the way of addressing pattern recognition or classification problems has evolved [21,22]. DL, together with the OpenCV Python Library, has been the tool of preference for several researchers when developing applications capable of understanding both facial and hand gestures. An example of the above is the study presented in [23], in which the translation of hand gestures combined with facial expressions is addressed with the objective of making communication more accurate. In the study, the authors concluded that a 97.96% success rate was obtained for the classification of two categories, i.e., classification of an input image between two emotions, namely “bored” and “astonished”. Meanwhile, in the study conducted in [24], a computer vision system is implemented, which can recognize signs from users and convert it into text in real time. To this end, sign gestures are captured and processed using the OpenCV Python library, after which the authors obtained a success rate of 99.91% in controlled light scenarios; however, this rate plummeted in environments with poor lighting. To tackle this issue, the authors proposed improving the computer vision system by including facial gestures to complement the information transmitted. In the same line, in [25], the results of multiple experiments are presented, with a success rate of 93.67% in controlled lighting scenarios, in which 90.04% corresponds to letters from the American sign alphabet, 93.44% to numbers, and 97.52% to static words. Other research has centered on the detection of human posture to subsequently classify gestures or expressions. In this line, the studies conducted in [26] present the novel design of a pipeline for the recognition of actions in videos recorded by a humanoid robot, which would give robots action recognition capacities to enhance social interaction. With this purpose, a sequence was created that employs a Common Spatial Patterns (CSP) algorithm that processes signals obtained from skeleton joints of the person performing the action on the video. Subsequently, a summary image was generated for each video, and classified using two different approaches, namely global visual descriptors and CNNs. This approach was first tested on two datasets that represent two scenarios with common characteristics, and then the results were compared through a Long Short-Term Memory (LSTM) method. Following this same line of work, the study in [27] describes how advances in artificial intelligence can be used to remove the communication barriers of the deaf community. To this end, using the MediaPipe library and an automatic learning algorithm, a methodology was developed that simplified the recognition of sign language. As a result, an accurate tracking of hand movements with different phalanx movements and deviations in the finger joints was achieved, with a 99% success rate, employing the Support Vector Machine (SVM) for real-time detection of hand signs, without portable sensors, which makes this technique easy and convenient. Meanwhile, in [28], the MediaPipe library and a hybrid CNN model with Bi-directional Long Short Term Memory (Bi-LSTM) was used for pose details extraction and text generation. Additionally, a hybrid Machine Translation (NMT) model, the MediaPipe library and Dynamic Generative Adversarial Network (GAN) were employed to obtain sign gesture videos for given spoken sentences. According to the results, the success rate was above 95%.

The study in [29] used rules based on angles and lines for the classification stage, efficiently increasing the general performance of machine learning algorithms. Hand gesture recognition was implemented in real-time; then, the full algorithm was tested in two known sign language datasets, namely the ASL alphabet (American Sign Language) and the ISL-HS (Irish Sign Language) sign language datasets. As a result, a 93% success rate was achieved with the ASL alphabet dataset, while a success rate over 96% was obtained with the ISL-HS dataset. However, there were limitations when dynamic datasets were included due to the wide variety of possible hand positions.

In [30], three customs datasets were created to train three different DL models employing the MediaPipe library. Two of these models were specifically used to predict groups of letters that are like one another, which solves the problem of similar signs in the same context. Additionally, an interface was developed to visualize the classification in real time.

In [31], an approach is proposed that comprises a palm recognition model and a linear recognition model to identify the numbers and letter of the fingerprint alphabet in the Kazajo sign language. This method was subject to experimental tests using a kinematic model of the hand and data obtained through a magnetic location system. These tests assessed the results of the letters in two different positions, obtaining a classification accuracy of approximately 97%.

In the studies conducted in [32,33], the usefulness and multifunctionality of the MediaPipe library is underscored, both for the study and evolution of Parkinson’s Disease (PD) and for the automated correction of human posture during heavy load work. Therefore, this demonstrates the validity of developing gesture recognition systems based on deep learning to improve processing time and response accuracy.

From the perspective of bioinspired models, various studies that reveal the benefits of their application are presented below.

In the study conducted in [34], the implementation of a robust retina bioinspired model is presented. This model was provided with feedback through information directly from the human brain in order to recognize the daily activities of a human, such as walking, running, and jumping. This type of work demonstrates the feasibility and potential of multiple combinations of brain signals. Other studies suggest different approaches or applications of bioinspired retina models. In turn, the study in [35] proposes a bioinspired system based on fish retinas to solve the problems of underwater image degradation due to blurring and nonuniform color biasing. The most relevant aspect of this model is its capacity to adapt to the environment, since it can operate without previous knowledge of water conditions. Additionally, a luminance-based fusion strategy was used to rebuild the enhanced image from the outputs of ON and OFF pathways of fish retinas. Meanwhile, the study conducted in [36] presents a theoretical analysis of the efficient coding of natural movies for the existence of the four most dominant cell types in the primate retina that together comprise 70% of all ganglion cells. The research in [37] addresses the design of a filter that allows the human retina to increase the sharpness of the visual stimulus before its brain transmission, thanks to which noticeable improvements are achieved in image processing.

In turn, the research in [14,15] shows that, with the support of retinal bioinspired models, Reinforcement Learning (RL) methods become fast and reliable for decision-making.

From a more general perspective, and with the purpose of recognizing movements related to recurring activities and body expressions, research such as [28] presents a model called DMLC-CNN for detecting and determining human activity in video sequences captured by a drone. The authors claim that the proposed system, after experimental evaluation, shows an accuracy of 94.50%, precision and recall of 94.50%, along with a sensitivity of 94.51%. Furthermore, the proposed model consumes less time (36.017 ms) for processing. When comparing these results to existing approaches, the proposed mechanism requires less training time. Similarly, in all metrics, the proposed approach outperforms conventional methods. However, the proposed model cannot predict human activities on water surfaces.

On the other hand, in research such as [29], studies on human gestures in sports are presented to analyze, guide, and evaluate activities. For this purpose, a combination of Long Short-Term Memory networks (LSTM) with a framework of Bio-Inspired Algorithms (BIA) is proposed to recognize actions and motivate skill improvement. Spatial Pyramid Pooling (SPP-net) is also incorporated to ensure robust feature extraction. The proposed approach achieves high accuracy in action recognition, motivating athletes to enhance their skills. Experimental results demonstrate the superior accuracy, precision, prediction, retrieval, and performance ratio of the proposed method compared to other techniques. Similarly, to the previous research but focusing on recognizing facial expressions [30], a new technique called Fusion-CNN is proposed to increase facial recognition accuracy even in cases of facial occlusion or changes in lighting conditions. This technique enhances accuracy by extracting hybrid features using an undirected β-skeleton graph and an ellipse with parameters trained using a 1D-CNN. Additionally, a 2D-CNN is trained on the same image. The outputs of these two subnetworks are fused, and their features are concatenated to create a feature vector for classification in a deep neural network. The performance of Fusion-CNN is compared with four public facial datasets, namely: the Cohn—Kanade dataset (CK+) extended dataset, the Japanese Female Facial Expression (JAFFE) dataset, Karolinska Directed Emotional Faces (KDEF), and Oulu-CASIA. Experimental results show that Fusion-CNN outperforms other algorithms, achieving recognition accuracies of 98.22%, 93.07%, 90.30%, and 90.13% for the CK+, JAFFE, KDEF, and Oulu-CASIA datasets, respectively. Therefore, it can be affirmed that bioinspired models improve various aspects of a scene, allowing the optimization of environmental perception for some agent or system. In this work, the implementation of bioinspired photoreceptor cells is chosen, since in conditions of high or low illumination the captured image is processed to self-regulate its illumination and consequently the contrast. This makes it possible to increase the probability of success in classification stages in real environments (scenarios with varying illumination).

Consequently, from the study of the state of the art, this work presents a convolutional neural network model, fed by bioinspired cells to increase accuracy in the classification process of a class.

## 3. Structure of the PhotoBiological Filter Classifier

Considering the current advances in research on this topic, this work proposes the design and implementation of a static gesture recognition system using bioinspired photoreceptor cells capable of self-regulating the dynamic lighting of the environment which is supported by the integration of the MediaPipe library to facilitate the detection of the human hand, classification through DL, and finally, the translation of sign language.

Specifically, the global architecture consists of four fundamental stages: the first is the self-regulation of the scene lighting by means of a bioinspired photoreceptor cell model, the second corresponds to hand detection anywhere in the scene, the third corresponds to the segmentation of the ROI, and the fourth corresponds to the classification of the gesture detected in the ROI.

**Figure 1:**
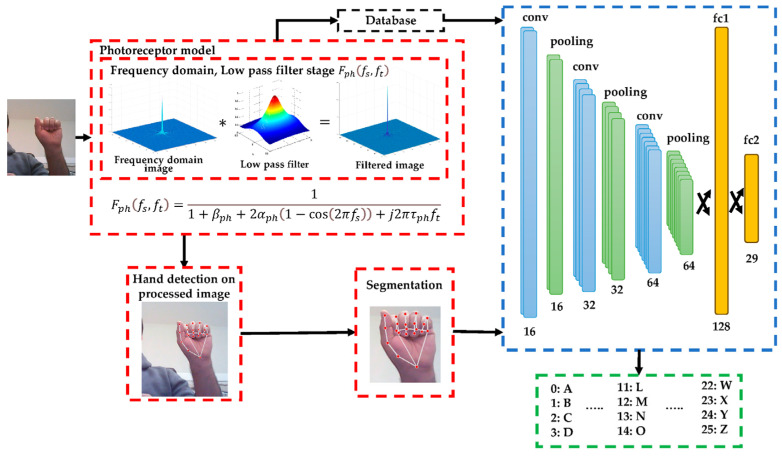
Block diagram of the system proposed where “*” represents that when an image is processed by a low pass filter, it results in a filtered image.

From these stages, a dataset for training and validation is preprocessed by photoreceptor cells before being applied to the model for training. Figure 1 shows a block diagram of the bioinspired model structure, which includes a DL model. All the steps involved in the system are described below.

### 3.1. Bioinspired Model and Retina Cells

**Figure 2:**
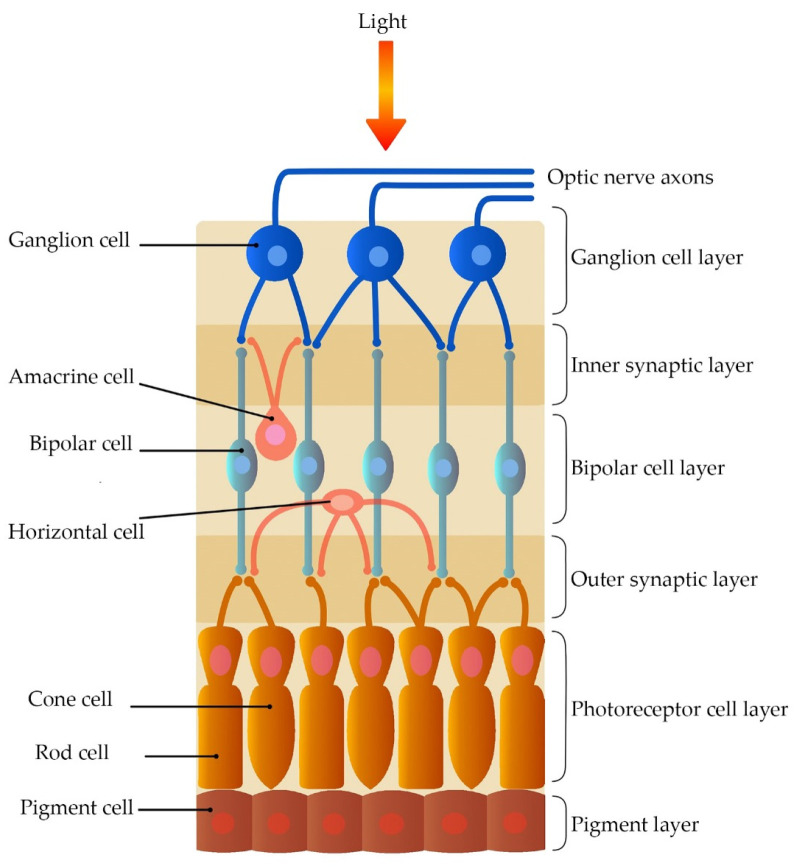
Representation of retinal cell communication during light absorption.

Once it enters the eye, light is absorbed in the Outer Plexiform Layer (OPL), composed of photoreceptors (cones and rods), as well as horizontal and bipolar cells. Subsequently, light enters the Inner Plexiform Layer (IPL), which comprises the response of the bipolar, amacrine, and ganglion cells (see Figure 2).

**Figure 3:**
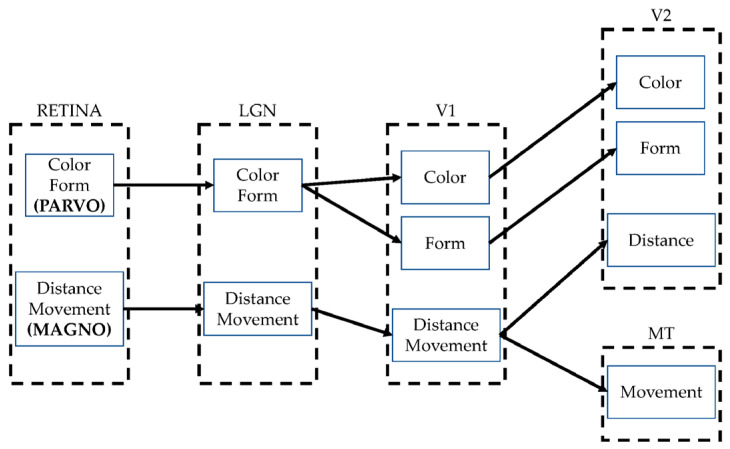
Summary of the parallel ways of visual processing.

Given that the outer and inner plexiform layers are a set of fused low pass and high pass filters that form a space-time filter, image processing is mainly conducted in the frequency domain, which facilitates obtaining the Parvocellular channel (Parvo) and the Magnocellular channel (Magno). Both ways are closely related, but each transports a specific aspect of the scene, as shown in Figure 3. The use of Parvo generates detail extraction, which responds to the question *what is this?* In turn, motion information extraction is obtained with Magno, which answers the question *where is it?* Both channels are connected to the Lateral Geniculate Nucleus (LGN) from which information is sent to the diverse areas corresponding to visual processing, namely V1, V2, V3, V4, and V5, among others [38,39].

### 3.2. Bioinspired Photoreceptor Model

The mathematical model for photoreceptor cells can be expressed through Equation (1), which clearly states that this model depends on spatial frequency $f_{s}$ and time frequency $f_{t}$, where $\beta_{ph}$ is the gain of $F_{ph}$, $\alpha_{ph}$ is the spatial filtering constant that allows for adjusting the cut-off value to the high frequencies, and $\tau_{ph}$ is a time filtering constant to minimize temporal noise. Therefore, the response of filter $F_{ph}$ corresponds to the response of the ON bipolar cells:

(1) $$F_{ph}\left(f_{s},f_{t}\right)=\frac{1}{1+\beta_{ph}+2\alpha_{ph}\left(1-\cos\left(2\pi f_{s}\right)\right)+j2\pi \tau_{ph}f_{t}}$$

**Figure 4:**
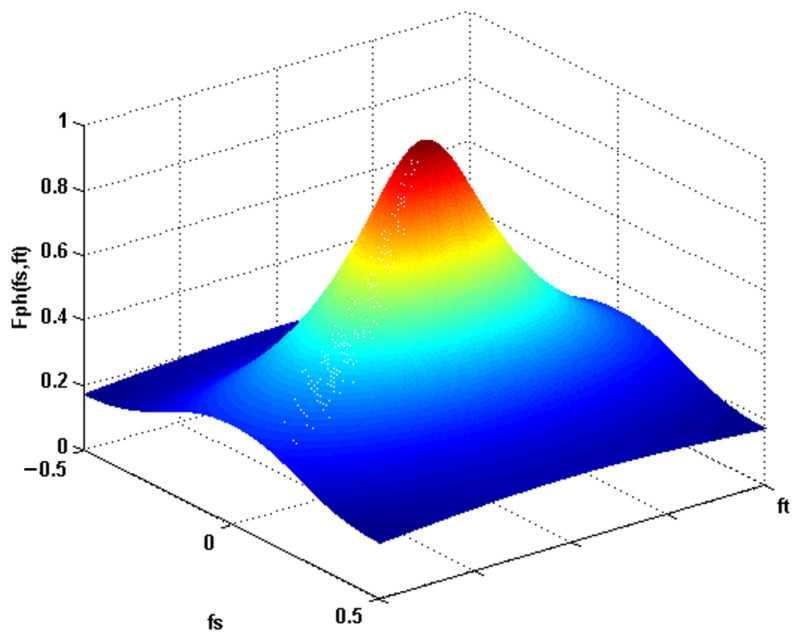
Response of the low pass filter.

Figure 4 shows the behavior of the photoreceptors in the frequency domain.

Therefore, it is stated that the photoreceptor cell model is considered as a low pass filter in the frequency domain. If we want to apply the filter to an RGB $\epsilon \;R^{3}$ image, whose spatial dimension is nxm, it must first be converted from the spatial domain to the frequency domain by means of the Fourier transform to decompose the image into a sum of sines and cosines. Mathematically, the Fourier transform is expressed by Equation (2), while Equation (3) indicates the Euler equation.

(2) $$F\left(k,l\right)=\overset{N-1}{\underset{i=0}{\sum }}\overset{N-1}{\underset{j=0}{\sum }}f\left(i,j\right)e^{-i2\pi \left(\frac{k_{i}}{N}+\frac{l_{j}}{N}\right)}$$

(3) $$e^{ix}=\cos\left(x\right)+isin\left(x\right)$$

From Equation (2) it follows that $f\left(i,j\right)$ is the value of the image in its spatial domain and $F\left(k,l\right)$ in its frequency domain. The result of the transformation is complex numbers. This represents a real image (magnitude image) and a complex image (phase image). However, only the magnitude image is of interest, since it contains all the information about the geometric structure of the image $f\left(i,j\right)$. However, both parts are necessary to return to the spatial domain.

It should be noted that digital images, being discrete, can take on a value from a given domain. For example, in an 8-bit grayscale image, the values are usually between 0 and 255. Therefore, the Fourier Transform also needs to be discrete, resulting in a Discrete Fourier Transform (DFT).

### 3.3. Light Regulation Model

Photoreceptors can adjust their sensitivity according to the light received from their surroundings. The model in Equation (4) has been adapted from the Michaelis—Menten model [38] to process images $\epsilon \;R^{2},$ since it is originally an equation ϵ R and allows for normalizing light ranges between 0 and $V_{max}$.

(4) $$C\left(m,n\right)=\frac{R\left(m,n\right)}{R\left(m,n\right)+R_{0}\left(m,n\right)}V_{max}+R\left(m,n\right)$$

where $R_{0}$ is defined by:

(5) $$R_{0}\left(m,n\right)=V_{0}L\left(m,n\right)+V_{max}\left(1-V_{0}\right)$$

Therefore, the adjustment to light $C\left(m,n\right)$ of a photoreceptor p located at the coordinate m,n is defined by current light brightness $R\left(m,n\right)$ and the compression parameter $R_{0}\left(m,n\right)$, which is directly linked to light in the vicinity $L\left(m,n\right)$ of the photoreceptor p located at the coordinates m,n and corresponds to the frequency response of the filter $F_{ph}$ (Equation (1)) on the image to be processed. In turn, the constant $V_{0}$ is a compression parameter that can take values between 0 and 1, while $V_{max}$ is the maximum value of the pixel to be processed.

**Figure 5:**
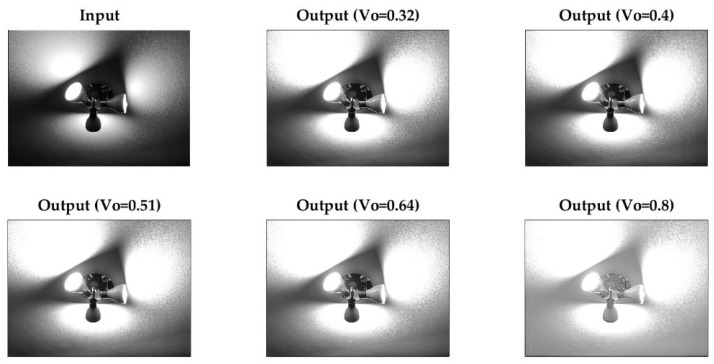
Response of the photoreceptor model to multiple values of $V_{0}$.

**Figure 6:**
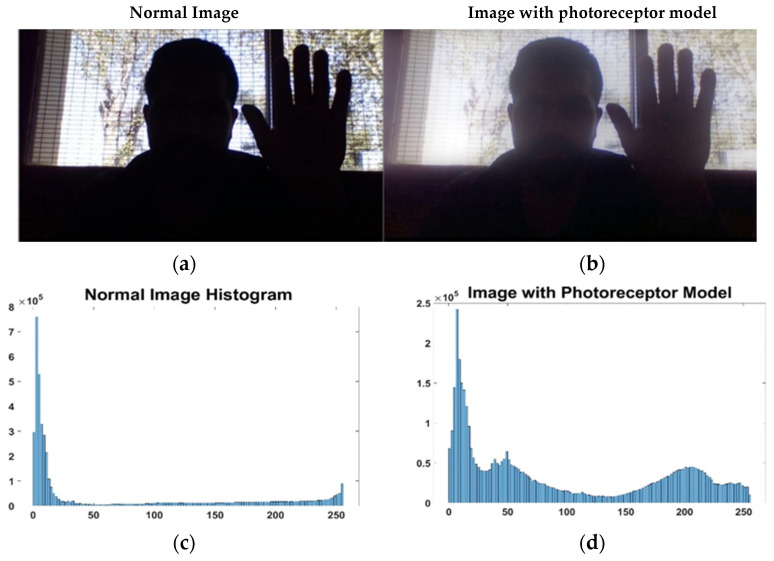
Example of response of the photoreceptor model with *V*_0_ = 0.7: (**a**) original image entering the system, (**b**) image processed by bioinspired photoreceptor cells, (**c**) histogram of image (**a**), and (**d**) histogram of image (**b**).

The mathematical model allows for modifying the brightness of the darker pixels while keeping lighter pixels constant or close to their value. To verify this effect, Figure 5 presents the response of the photoreceptor system to multiple values of $V_{0}$. Figure 6 below shows the effect of the response of the photoreceptor system (with $V_{0}=0.7$) in an extreme unfavorable case since dark tones tend to predominate in the image (normal image).

In detail, Figure 6 shows a comparison of an image taken in a daily environment, (a), with the same image, but processed by bioinspired photoreceptor cells, (b). It should be noted that image (a) limits correct operation in most artificial vision systems. In fact, most of the facial features are not appreciated and are confused with elements of the scene. In terms of histograms, (c) presents an imbalance, since it is appreciated how a large number of pixels are distributed around the values 0 and 30, that is, very dark tones, while the histogram of the image processed by photoreceptor cells, (d), presents a more homogeneous distribution, considerably reducing the dark tones. This homogeneous distribution of the histogram allows recovering information evident in (b) such as facial features and skin tone, as well as garments such as the jacket.

In summary, Equation (4) acts as a real-time light regulator to regulate and balance the brightness of a scene. That is, it slightly enhances the white areas of the environment, while amplifying the low-brightness areas of the scene to a greater extent. It is hoped that this allows for a balanced histogram, highlighting areas that were previously not easily visible.

### 3.4. Application of MediaPipe Library

**Figure 7:**
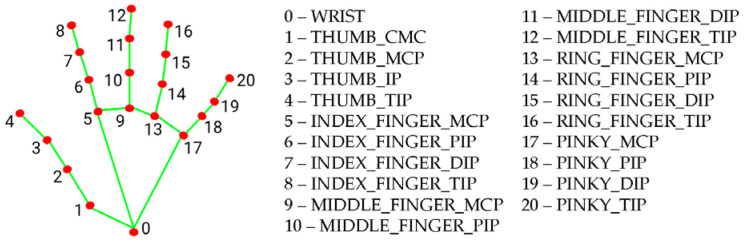
Representation of the 21 points detected by the MediaPipe library on the hand.

MediaPipe is a multiplatform library developed by Google that provides automatic learning solutions for computer vision tasks using only a regular webcam. When the MediaPipe library is used on hands, it is possible to detect and extract the coordinates of its 21 joints in a 3D environment, as shown in Figure 7.

**Figure 8:**
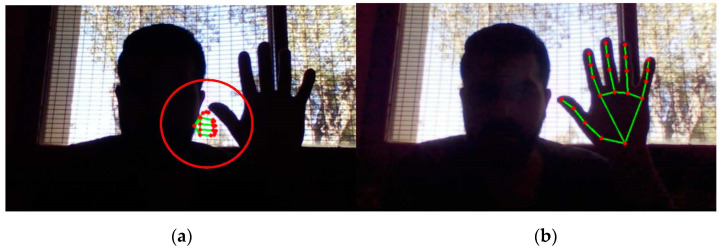
Extraction of the 21 points of interest: (**a**) original image with failed hand detection by MediaPipe library and (**b**) image processed by bioinspired photoreceptor cells with successful hand detection by MediaPipe library.

The MediaPipe library is integrated into this research to easily segment the ROI and generate the classification process in a simple way. Although the MediaPipe library demonstrates great real-time performance, it is still conditioned by environmental variables such as lighting. In fact, in Figure 8a, it can be seen how poor lighting generates a poor result for detecting the location of the hand (red circle), while in Figure 8b, the correct detection of the hand location is observed, in the same scene, but processed by photoreceptor cells.

### 3.5. Application of the Deep Learning Model

Deep Learning allows for the classification of an object detected in a scene through the use of CNNs. The stages of the DLM are described below.

#### 3.5.1. Training and Validation Database

**Figure 9:**
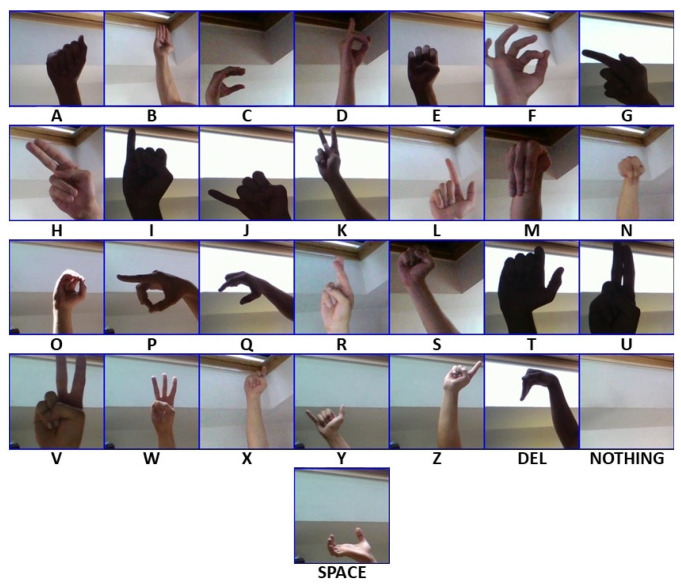
Collection of samples per class from dataset [16].

There is a wide variety of online datasets to solve different cases of interest, such as detection and classification of objects, numbers, colors, or any specific pattern. This research addresses the classification of American sign language, but focused on the letters of the alphabet. For this reason, use is made of [16] which claims to have 87,000 images which are 200 × 200 pixels. There are 29 classes, of which 26 are for the letters A–Z and three classes are for SPACE, DELETE, and NOTHING. Figure 9 shows a sample per class of said dataset.

**Figure 10:**
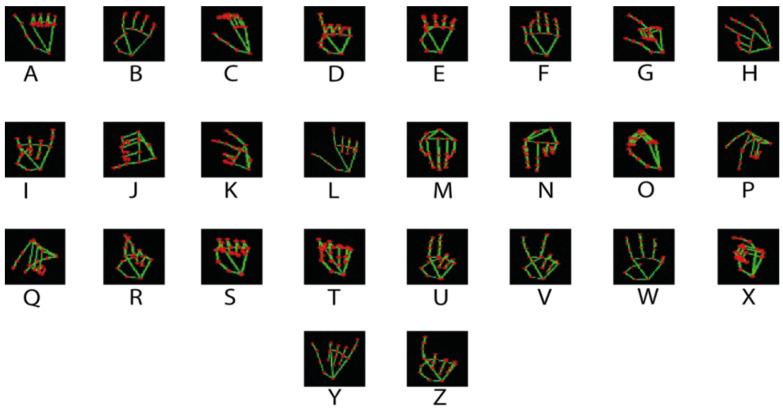
Representation of the dataset used for the training and validation of the system.

It should be noted that from the dataset of [16], two additional datasets are generated, which will allow training three different models, the first being trained with the dataset without prior processing, the second model being trained with images processed by bioinspired photoreceptor cells, and the third model being trained with a dataset obtained from the images processed by photoreceptor cells, and subjected to the MediaPipe library in order to extract the landmark of the hand position which contains the 21 points of the joint. Figure 10 exemplifies the third dataset to be used.

#### 3.5.2. Deep Learning Model

**Figure 11:**
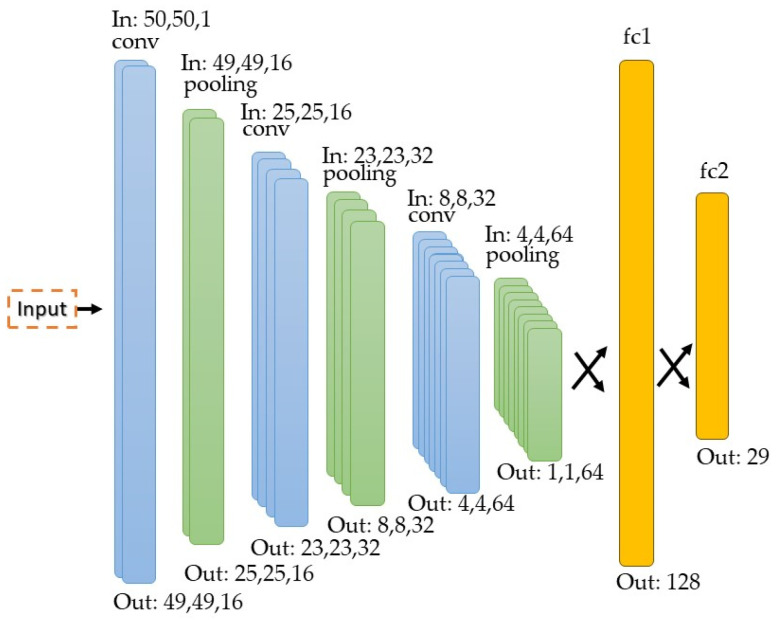
Structure of the neural network.

The developed model has a simple structure capable of recognizing several classes. In this case, 29 different classes are used, of which 26 correspond to the letters of the American alphabet and the other three correspond to the “delete”, “nothing”, and “space” classes. This model is composed of 16 filters in the first convolutional layer with a 2 × 2 kernel, and a pooling layer that reduces the spatial dimension to 25 × 25 × 16. The next convolutional layer has 32 filters with a 3 × 3 kernel, and the pooling filter reduces the spatial dimension to 8 × 8 × 32. The last convolutional layer has a 5 × 5 kernel and 64 filters, while the last pooling filter reduces the spatial dimension to 1 × 1 × 64. Finally, there is the fully connected stage fc1 and fc2 that has 29 different output classes. The model uses a ReLU activation function in each layer and a SoftMax activation function to predict the corresponding category. Finally, it is important to point out that there are 68,045 parameters to be trained. Figure 11 presents the neural network structure.

## 4. Development

The implementation of the bioinspired photoreceptor cell system is conducted on a desktop whose main features are the following: Intel core i7, 16GB RAM, AMD Radeon M275, and 1024 × 728 resolution webcam (Logitech C170, Lausanne, Switzerland.).

### 4.1. Quantitative Analysis of the Model

Using the scikit-plot library it is possible to generate a model evaluation report that, through a table of precision, recall, F1 score, and support, provides a quantitative report. As a whole, these specifications express the performance of the model in real time when making the recognition. The precision column indicates the proportion between the True Positive (TP) predictions and the total positive predictions (see Equation (6)), while the recall column indicates the proportion between the true positive predictions and the total positive instances (see Equation (7)). On the other hand, F1 score is a measure that relates precision and recall and corresponds to a metric that takes into account both False Positives (FP) and False Negatives (FN) (see Equation (8)). Finally, support corresponds to the number of instances of each class in the dataset; it is simply the total number of examples that belong to a specific class and is used to obtain an idea of how many real elements exist for each class and allows interpreting the other metrics in their context.

(6) $$\mathrm{Precision}=\frac{\mathrm{TP}}{\mathrm{TP}+\mathrm{FP}}$$

(7) $$\mathrm{Recall}=\frac{\mathrm{TP}}{\mathrm{TP}+\mathrm{FN}}$$

(8) $$F1-\mathrm{Score}=\frac{2\cdot \left(\mathrm{Precision}\cdot \mathrm{Recall}\right)}{\mathrm{Precision}+\mathrm{Recall}}$$

Additionally, another important parameter is accuracy, since, in relation to the total number of prediction cases, it shows the number of successful cases when testing the model (see Equation (9)).

(9) $$\%\;\mathrm{Accuracy}\;=\frac{\mathrm{No}.\;\mathrm{successful}\;\mathrm{recognition}\;\mathrm{cases}}{\mathrm{No}.\;\mathrm{total}\;\mathrm{cases}}$$

Additionally, a successful recognition case is that in which the user generates a sign, and this is correctly translated by the system within 15 s. In case the gesture fails to be recognized, it is considered an error case.

### 4.2. Parameters of the Bioinspired Model

The parameters used in the bioinspired photoreceptor model were selected heuristically, obtaining an optimal performance. Table 1 shows the employed parameters.

### 4.3. Test Results

As mentioned above, tests are carried out with three datasets that arise from dataset [16]. Each version of the model to be tested with a dataset will be identified as V1, V2, and V3, with V1 being the tests carried out with the original dataset, V2 the tests on the dataset processed by retinal cells, and V3 the tests on the dataset processed by retinal cells and the MediaPipe library.

Table 2 shows in detail the results obtained for “Precision”, “Recall”, “F1-Score”, and “Support” for V1, V2, and V3. Additionally, the column “% accuracy” is incorporated, which corresponds to the empirical results of the system on 100 tests for each of the 29 classes.

For the case of V1, the letters that present the worst performance (below 50%) are “A”, “B”, “H”, “N”, “R”, “S”, and “Z”. These same ones in V2 present a general improvement; for example, for the letter “A”, the accuracy improves from 30 to 42, while for the case “B”, there is a limited improvement from 35 to 37. Globally, V2 presents 3.1 more points of accuracy with respect to V1. From the point of view of V3, having a reinforcement of photoreceptor cells and 21 articulation points (MediaPipe), there is an overall improvement of 24.5 more points of accuracy compared to V2 and 27.7 more points of accuracy compared to V1. Regarding the Frame Per Second (FPS) rate, the system consistently reaches 55 FPS for V1, V2, and V3.

In general, it is observed that, although the improvement corresponds only to the use of MediaPipe, it is necessary to consider that the photoreceptor cells are the ones contributing to the detection performance of MediaPipe in V3. Therefore, it is the contribution of both elements that enables this result.

### 4.4. Performance Tests under Different Training Networks

Next, and to complement the results provided in Table 2, an additional experiment is presented where the V1 and V2 datasets are tested under the YoloV3, YoloV5, and YoloV8 architectures.

In the case of the previously presented model, a grayscale dataset with dimensions of 50 × 50 is utilized, while the Yolo family requires an RGB input set of 640 × 576 pixels. Therefore, the original dataset (200 × 200 pixels) is resized.

Table 3 shows the comparison between YoloV3, YoloV5, and YoloV8 regarding the accuracy achieved when applying photoreceptor cells and in their absence. This demonstrates that the overall improvement trend is maintained with the simple action of processing the training images with bioinspired photoreceptor cells.

From the perspective of the obtained FPS, considering that V1, V2, and V3 models operate at a higher image rate (55 FPS), these models are 27.5 times higher than the FPS of the YoloV3 model, 30.5 times higher than the FPS of the YoloV5 model, and 40.3 times higher than the FPS of the YoloV8 model. This difference in FPS is due to the inherent architecture of Yolo, which has been designed to classify a large number of classes at higher resolutions.

### 4.5. Comparative Analysis

The results obtained in this work (letter recognition and translation) are compared with the results of other similar research that uses 2D image processing and uses the American Sign Language. Table 4 summarizes this comparison and expresses the structure or configuration used, the accuracy, and the year of publication.

From Table 4, the following can be mentioned:

- The authors of [25] introduce a static sign recognition model (2D) tested in three experiments. In the first experiment, the American alphabet is recognized; in the second, the first 10 numbers are recognized; and in the third, a total of 34 gestures are recognized. As a result, the accuracy of recognizing the American alphabet is 90.04%, but with a processing time of approximately 4 s per result. In contrast, our system demonstrates both higher accuracy and improved processing time;
- The authors of [17] introduce an implementation of a real-time processing device based on the YoloV3 architecture, which allows recognizing the alphabet and a set of gestures at 48 FPS with an accuracy of 90.77. However, our proposed model is able to work at 55 FPS;
- The authors of [29] introduce a gesture classification based on the angles formed by the keypoints obtained from MediaPipe. In this way, they generate models by Decision Tree, Random Forest, and Naïve Bayes, obtaining the best accuracy by Random Forest with 93%, but in a controlled lighting environment. However, our proposed model allows working in environments with dynamic and bounded lighting;
- The authors of [30] introduce a CNNs model fed with a dataset of the distances between the keypoints extracted by MediaPipe, thus allowing 94% precision when classifying the American alphabet. In this regard, our proposed model differs in the dataset, since the model is fed directly with the landmarks extracted from MediaPipe;
- Finally, the authors of [27] introduce MediaPipe-based training for both 2D and 3D processing, but they are limited only to the first 10 numbers.

## 5. System Limitations and Its Adaptation to Lighting Conditions

The proposed system can adapt to different lighting conditions, and after several performance tests, it is demonstrated that between 30 to 50 minimum lumens (around the hand) are sufficient to achieve hand recognition. Furthermore, it is worth noting that the system has been trained for recognizing one gesture at a time; however, this does not prevent it from being able to detect more than one hand in its environment. In the conducted tests, the detection of two hands simultaneously in real time has been achieved, and when a third hand is introduced, the system ignores it. Additionally, the system is capable of processing both images and real-time video. When it detects one of the possible letters of the alphabet, the display will indicate that gesture and will only be updated if a new alphabet-specific gesture is entered. Thus, regardless of whether an existing gesture in the dataset is performed, the MediaPipe library will track the hand at all times.

Although this system has been trained with 29 different classes for one hand, it allows for adding new classes to enable more complex classifications, such as gestures involving two hands simultaneously.

## 6. Conclusions

In this work, the design and implementation of a novel PhotoBiological Filter Classifier applied for the recognition and translation of static sign language is presented.

To validate the advantages of applying photoreceptor cells, 100 tests were conducted for each letter to be recognized, resulting in an average accuracy of 91.1% for V3, compared to 63.4% obtained for V1. The average classification iteration time for each letter was 55.5 FPS for V1, V2, and V3, demonstrating that the use of photoreceptor cells does not impact processing time.

The V3 system requires fewer epochs compared to V2 and V1. This is due to the nature of the generated dataset, allowing for minimal weight adjustment during training, unlike the case of V1.

As in any artificial vision application, the light of the scene should not be too high or low as this directly affects information capture and processing. Therefore, the use of the bioinspired photoreceptor model created allowed for rapidly regulating the light in the scene and, consequently, its contrast.

The designed and implemented system primarily showcases the advantages of integrating bioinspired models into systems interacting with the environment. This not only enhances decision-making in reinforcement learning but also improves accuracy percentages in deep learning. Furthermore, the computational resources required by a bioinspired model are relatively low.

Unlike other works, this research designs and implements a bioinspired photoreceptor model that utilizes landmark segmentation on a black background, extracted by MediaPipe. This approach allows for generalizing a dataset and achieving high accuracy with low processing time.

In the field of gesture translation, the HSV color range (Hue, Saturation, Value) used to be employed as a filter to facilitate the recognition of bare hands, capturing skin tone areas. However, the use of an HSV model poses a problem when recognizing areas with colors similar to skin. Thanks to deep learning, various research now directly uses RGB scenes to feed some CNN models. Although overall results improve, processing time remains high. Therefore, the application of the MediaPipe library has become popular to segment the ROI and improve classification times and accuracy. This research contributes to the development and deepens the use of bioinspired photoreceptor cells with the MediaPipe library for training a deep learning model, demonstrating improvements in accuracy percentage with an average time of 20 milliseconds compared to models without photoreceptor cells.

## 7. Future Work

Given the proven advantages of the application of photoreceptor cells, the implementation of parvocellular cells to highlight contours in the scene, as well as the incorporation of magnocellular cells to detect movements in the scene, are foreseen.

All of the above aims to generate a dynamic sign translator, i.e., to analyze the dynamics of an object over time based on a set of captured images. In this way, it will be possible to conduct performance comparisons with respect to various cutting-edge works presented in the state of the art.

Moreover, the use of the MediaPipe library is proposed for the detection of facial expressions and body movements to include more variables in the gesture classification process.

Finally, following this work, we expect to develop a downloadable version for smartphones. This version, in addition to being a gesture classifier, will allow for providing feedback to the new model to expand its database.

## Figures and Tables

**Table 1:** Parameters entered.

**Light Regulator**	
*V*_0_	0.7
*V_max_*	255
**Filter *F_ph_***	
*β_ph_*	0
*α_ph_*	1
*τ_ph_*	1

**Table 2:** Result obtained by V1 version.

Letter	Precision			Recall			F1-Score			Support			% Accuracy		
	V1	V2	V3	V1	V2	V3	V1	V2	V3	V1	V2	V3	V1	V2	V3
A	0.99	0.98	0.99	1.00	0.96	0.98	0.99	0.97	0.98	237	254	205	30	42	92
B	1.00	0.99	0.99	0.99	1.00	0.99	1.00	0.99	0.99	257	265	182	35	37	95
C	1.00	0.99	0.99	1.00	1.00	1.00	1.00	0.99	1.00	246	257	169	80	80	99
D	1.00	0.99	0.99	1.00	1.00	0.99	1.00	0.99	0.99	264	242	243	75	72	95
E	0.99	0.98	0.97	1.00	0.98	1.00	0.99	0.98	0.98	246	247	190	60	70	88
F	1.00	1.00	0.99	1.00	1.00	1.00	1.00	1.00	0.99	241	251	255	55	57	85
G	1.00	0.99	1.00	0.99	0.99	1.00	0.99	0.99	1.00	247	259	232	62	65	92
H	0.99	1.00	1.00	1.00	0.99	1.00	0.99	0.99	1.00	250	251	220	45	45	85
I	1.00	0.98	0.99	0.99	0.98	0.99	1.00	0.98	0.99	275	247	202	75	77	92
J	1.00	0.99	0.99	1.00	0.99	0.99	1.00	0.99	0.99	246	259	214	82	82	95
K	0.99	0.98	1.00	0.99	0.98	0.99	0.99	0.98	1.00	237	260	249	75	76	96
L	1.00	1.00	0.99	1.00	1.00	1.00	1.00	1.00	0.99	263	250	246	90	95	99
M	1.00	0.95	0.92	1.00	0.97	0.91	1.00	0.96	0.92	218	235	154	55	58	89
N	1.00	0.97	0.94	1.00	0.97	0.91	1.00	0.97	0.92	212	264	127	40	55	85
O	1.00	1.00	0.97	0.99	0.98	0.98	1.00	0.99	0.98	268	253	203	85	87	92
P	1.00	1.00	0.98	1.00	0.99	0.99	1.00	0.99	0.98	265	269	225	53	52	78
Q	1.00	0.99	0.99	1.00	1.00	0.97	1.00	0.99	0.98	248	268	187	65	67	85
R	1.00	0.98	0.97	0.98	0.96	0.98	0.99	0.97	0.97	269	256	213	32	38	82
S	1.00	0.96	0.99	0.99	0.96	0.98	0.99	0.96	0.98	266	227	224	40	42	84
T	1.00	0.98	0.99	1.00	0.99	0.97	1.00	0.98	0.98	245	268	195	60	63	92
U	0.98	0.96	0.99	0.99	0.96	0.97	0.99	0.96	0.98	251	229	194	92	95	98
V	0.98	0.96	0.97	0.99	0.98	1.00	0.99	0.97	0.98	238	258	208	80	83	92
W	1.00	1.00	0.99	1.00	0.98	0.99	1.00	0.99	0.99	248	242	202	70	77	99
X	0.99	0.99	0.98	0.99	0.97	0.98	0.99	0.98	0.98	250	236	194	60	67	87
Y	1.00	0.98	0.99	1.00	1.00	1.00	1.00	0.99	0.99	239	216	216	95	95	99
Z	0.99	1.00	0.99	1.00	0.99	0.99	1.00	0.99	0.99	259	248	202	42	42	89
Delete	1.00	1.00	0.99	0.99	1.00	0.98	1.00	1.00	0.99	264	272	186	60	62	91
Nothing	1.00	1.00	-	1.00	1.00	-	1.00	1.00	-	239	254	-	90	92	-
Space	1.00	1.00	0.98	1.00	1.00	0.97	1.00	1.00	0.98	262	213	153	55	55	95
Accuracy							1.00	0.98	0.98	7250	7250	5690	63.4	66.5	91.1
Macro Avg.	1.00	0.98	0.98	1.00	0.98	0.98	1.00	0.98	0.98	7250	7250	5690			
Weighted Avg.	1.00	0.98	0.98	1.00	0.98	0.98	1.00	0.98	0.98	7250	7250	5690			

**Table 3:** Recognition test result for different models with and without PhBFC.

Model	FPS	mAP(%)	RMS Accuracy (%) Without PhBFC	RMS Accuracy (%) With PhBFC
YoloV3	28	76	81	84
YoloV5	25	80	85	87
YoloV8	15.2	96	92	94

**Table 4:** Comparative analysis of this work and others (2D image processing).

Sign Language	Authors	Structure	FPS	% Accuracy	Year
ASL	[25]	Only CNNs	1	90.04	2021
ASL	[17]	MobileNet YoloV3 model	48	90.77	2022
ASL	[29]	Angles of keypoints from MediaPipe with ML	-	93.7	2022
ASL	[30]	Only CNNs	-	94.07	2022
Numbers	[27]	MediaPipe with Feedforward neural network	-	99	2023
ASL	V1	Only CNNs	55	63.4	2023
ASL	V2	Photoreceptor cell with CNN	55	66.5	2023
ASL	V3	Photoreceptor cell with CNN+MediaPipe	55	91.1	2023

## Data Availability

Data are contained within the article.
